# Bis[4-chloro-*N*′-(2-pyridyl­methyl­idene)benzohydrazidato]cobalt(III) nitrate sesquihydrate

**DOI:** 10.1107/S160053681004612X

**Published:** 2010-11-13

**Authors:** Dayu Wu, Yan Zhao, Hui Ye, Genhua Wu

**Affiliations:** a Anhui Key Laboratory of Functional Coordination Compounds, School of Chemistry and Chemical Engineering, Anqing Teachers College, Anqing 246011, Anhui, People’s Republic of China

## Abstract

In the title compound, [Co(C_13_H_9_ClN_3_O)_2_]NO_3_·1.5H_2_O, the central Co^3+^ atom in the cation is coordinated by four N and two O atoms from the two tridentate ligands in a distorted octa­hedral fashion. In the crystal, the cobalt complex cations are linked to the half-occupied and the fully occupied water mol­ecules, and the nitrate anion *via* classical inter­molecular O—H⋯O and O—H⋯N hydrogen bonds and weak C—H⋯O contacts.

## Related literature

For the structure of bis­{4-chloro-*N*′-[phen­yl(2-pyrid­yl)methyl­idene]benzohydrazidato}cobalt(III) nitrate methanol disolvate, see: Wu *et al.* (2010 ▶). For a related mononuclear cobalt compound, see: Herchel & Boca (2005 ▶) and for a bimetallic dicobalt(II) complex, see: Gavrilova *et al.* (2002 ▶). For related structures containing hydrazide groups, see: Liu *et al.* (2006 ▶); Cao *et al.* (2009 ▶).
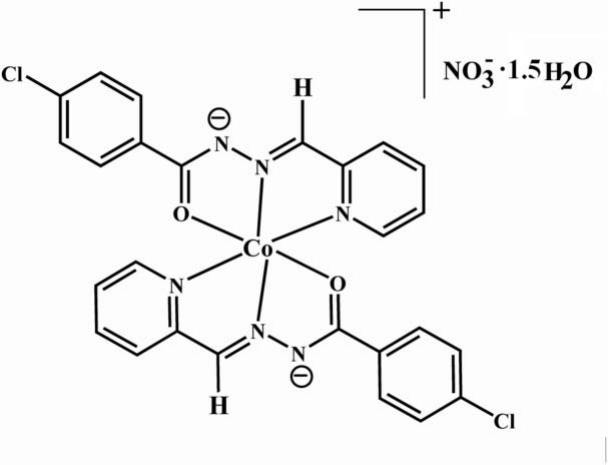


## Experimental

### 

#### Crystal data

[Co(C_13_H_9_ClN_3_O)_2_]NO_3_·1.5H_2_O
*M* *_r_* = 665.33
Monoclinic, 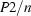

*a* = 14.198 (10) Å
*b* = 10.876 (7) Å
*c* = 18.553 (13) Å
β = 94.196 (12)°
*V* = 2857 (3) Å^3^
*Z* = 4
Mo *K*α radiation
μ = 0.84 mm^−1^
*T* = 293 K
0.32 × 0.22 × 0.18 mm

#### Data collection

Bruker SMART CCD area-detector diffractometer
Absorption correction: ψ scan (*SADABS*; Bruker, 1997 ▶) *T*_min_ = 0.765, *T*_max_ = 0.862
13702 measured reflections
5019 independent reflections
3527 reflections with *I* > 2σ(*I*)
*R*_int_ = 0.042

#### Refinement

*R*[*F* ^2^ > 2σ(*F* ^2^)] = 0.055
*wR*(*F* ^2^) = 0.163
*S* = 1.05
5019 reflections
388 parameters
5 restraints
H-atom parameters constrained
Δρ_max_ = 0.70 e Å^−3^
Δρ_min_ = −0.45 e Å^−3^

### 

Data collection: *SMART* (Bruker, 1997 ▶); cell refinement: *SAINT* (Bruker, 1997 ▶); data reduction: *SAINT*; program(s) used to solve structure: *SHELXS97* (Sheldrick, 2008 ▶); program(s) used to refine structure: *SHELXL97* (Sheldrick, 2008 ▶); molecular graphics: *SHELXTL* (Sheldrick, 2008 ▶); software used to prepare material for publication: *SHELXL97* and *PLATON* (Spek, 2009 ▶).

## Supplementary Material

Crystal structure: contains datablocks I, global. DOI: 10.1107/S160053681004612X/si2290sup1.cif

Structure factors: contains datablocks I. DOI: 10.1107/S160053681004612X/si2290Isup2.hkl

Additional supplementary materials:  crystallographic information; 3D view; checkCIF report

## Figures and Tables

**Table 1:** Selected bond lengths (Å)

Co1—N2	1.850 (3)
Co1—N5	1.855 (3)
Co1—O2	1.899 (3)
Co1—O1	1.914 (3)
Co1—N4	1.926 (4)
Co1—N1	1.931 (3)

**Table 2:** Hydrogen-bond geometry (Å, °)

*D*—H⋯*A*	*D*—H	H⋯*A*	*D*⋯*A*	*D*—H⋯*A*
O6—H61⋯O5	0.85	2.19	2.964 (8)	151
O6—H62⋯O7	0.85	2.09	2.804 (16)	141
O7—H72⋯N6^i^	0.85	2.32	3.053 (12)	145
C14—H14*A*⋯O4^ii^	0.93	2.41	3.229 (7)	146
C17—H17*A*⋯O5^iii^	0.93	2.46	3.263 (7)	145

Symmetry codes: (i) 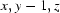; (ii) 
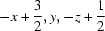
; (iii) 
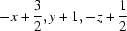
.

**Fig. 1.**
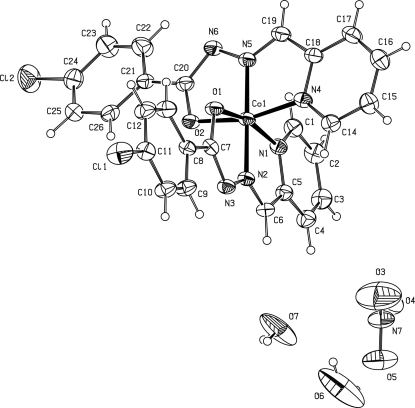
The molecular structure of the title compound. The thermal ellipsoids were drawn at 30% probability level using PLATON (Spek, 2009).

**Fig. 2.**
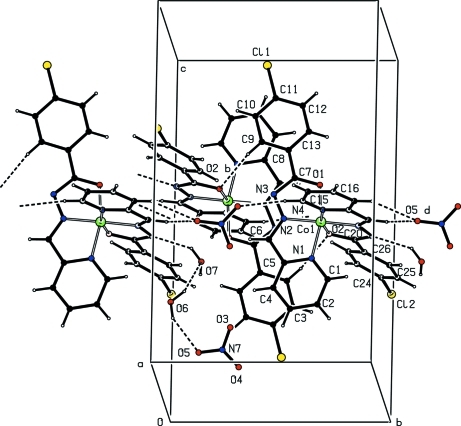
A section of the crystal packing with the hydrogen bonding indicated as dashed lines.

## supplementary crystallographic information

### Comment

We used a new ligand, 4-chloride-benzoylcarbohydrazide, to synthesize the title cobalt(III) complex (Bis[4-chloride-benzoylcarbohydrazido] cobalt(III) nitrate sesquihydrate). A related ethanol disolvate structure was recently published where we focussed on magnetic properties for this kind of cobalt(III) complex (Wu *et al.*, 2010). As a part of our ongoing investigations in this field we have synthesized the title compound and present its crystal structure here. For the title compound, we used 2(*E*)-1-[(4-chlorophenyl)carbonyl]-2-[(pyridin-2-yl)methylidene] diazanide as ligand, a typical rigid tridentate donor to synthesize a mononuclear compound, and we report the crystal structure of the complex [Co(C_13_H_9_N_3_OCl)_2_]^+^(NO_3_)^-^ 1.5(H_2_O) (Fig. 1). The coordination environments of Co(III) ions are completed by two ligands with average Co—N bond length of 1.891 Å and Co—O 1.907 Å (Table 1). The ligands adopt almost planar configurations, which are similar to those of two recently published hydrazide structures (Liu *et al.*, 2006 and Cao *et al.*, 2009).

In the crystal, the cobalt complexes are linked through the half-occupied, the full occupied water molecules and the nitrate anion *via* classic intermolecular O—H···O and O—H···N hydrogen bonds and weak C—H···O hydrogen bonding contacts (Table 2, Fig. 2).

### Experimental

Preparation of ligand: To the methanol solution of 4-Chlorobenzoic hydrazide (10 mmol, 1.7g) was added dropwise 2-pyridylcarboxylate (10 mmol, 1.1g) after stirring at boiling temperature for 1 hour, the white precipitate formed, which was filtered and dried over P_2_O_5_ in vacuum. (yield: 78%).Anal calc (%). for C_13_ H_10_ Cl_1_ N_3_ O: H 3.88 C 60.13 N 16.18. Found: H 3.76 C 60.34 N 16.87. Preparation of Co(III) complex: A methanolic solution (25 ml) containing the ligand (0.2 mmol, 0.052 g) was added dropwise to Co(NO_3_)_2_ 6 H_2_O (0.1 mmol, 0.029 g). After stirring for 15 minutes, the dark solution was filtered. Red block-shaped crystals suitable for single-crystal X-ray diffraction were obtained by evaporating the resulting filtration in air for several days (yield: 54.4%). Anal calc (%). for C_26_ H_22_ Cl_2_ Co N_7_ O_7_: H 3.29 C 46.36 N 14.56. Found: H 3.21 C 46.46 N 14.95.

### Refinement

C-bound H atoms were placed geometrically and allowed to ride during refinement with C—H = 0.93 Å with *U*_iso_(H) = 1.2 *U*_eq_(C). The water H atoms were located in a difference Fourier map and refined using distance restraints d(O—H) = 0.85 (1) Å and finally refined as riding with the parent atom with *U*_iso_(H) = 1.2*U*_eq_(O).

### Crystal data

**Table:**

[Co(C_13_H_9_ClN_3_O)_2_]NO_3_·1.5H_2_O	*F*(000) = 1356
*M**_r_* = 665.33	*D*_x_ = 1.547 Mg m^−^^3^
Monoclinic, *P*2/*n*	Mo *K*α radiation, λ = 0.71073 Å
Hall symbol: -P 2yac	Cell parameters from 5210 reflections
*a* = 14.198 (10) Å	θ = 2.5–50.3°
*b* = 10.876 (7) Å	µ = 0.84 mm^−^^1^
*c* = 18.553 (13) Å	*T* = 293 K
β = 94.196 (12)°	Block, dark-red
*V* = 2857 (3) Å^3^	0.32 × 0.22 × 0.18 mm
*Z* = 4	

### Data collection

**Table:**

Bruker SMART CCD area-detector diffractometer	5019 independent reflections
Radiation source: fine-focus sealed tube	3527 reflections with *I* > 2σ(*I*)
graphite	*R*_int_ = 0.042
φ and ω scans	θ_max_ = 25.0°, θ_min_ = 1.8°
Absorption correction: ψ scan (*SADABS*; Bruker, 1997)	*h* = −16→16
*T*_min_ = 0.765, *T*_max_ = 0.862	*k* = −12→10
13702 measured reflections	*l* = −22→18

### Refinement

**Table:**

Refinement on *F*^2^	Primary atom site location: structure-invariant direct methods
Least-squares matrix: full	Secondary atom site location: difference Fourier map
*R*[*F*^2^ > 2σ(*F*^2^)] = 0.055	Hydrogen site location: inferred from neighbouring sites
*wR*(*F*^2^) = 0.163	H-atom parameters constrained
*S* = 1.05	*w* = 1/[σ^2^(*F*_o_^2^) + (0.0924*P*)^2^ + 0.4119*P*] where *P* = (*F*_o_^2^ + 2*F*_c_^2^)/3
5019 reflections	(Δ/σ)_max_ < 0.001
388 parameters	Δρ_max_ = 0.70 e Å^−^^3^
5 restraints	Δρ_min_ = −0.45 e Å^−^^3^

### Special details

**Table:**

Experimental. The water oxygen atom O7 showed an approximate double value of the isotropic displacement parameter of water oxygen O6 (0.347 vs. 0.158). Therefore we set the site occupancy of O7 to 1/2 and refined the solvent water and the nitrate anion with anisotropic displacement parameters.
Geometry. All esds (except the esd in the dihedral angle between two l.s. planes) are estimated using the full covariance matrix. The cell esds are taken into account individually in the estimation of esds in distances, angles and torsion angles; correlations between esds in cell parameters are only used when they are defined by crystal symmetry. An approximate (isotropic) treatment of cell esds is used for estimating esds involving l.s. planes.
Refinement. Refinement of F^2^ against ALL reflections. The weighted R-factor wR and goodness of fit S are based on F^2^, conventional R-factors R are based on F, with F set to zero for negative F^2^. The threshold expression of F^2^ > 2sigma(F^2^) is used only for calculating R-factors(gt) etc. and is not relevant to the choice of reflections for refinement. R-factors based on F^2^ are statistically about twice as large as those based on F, and R- factors based on ALL data will be even larger.

### Fractional atomic coordinates and isotropic or equivalent isotropic displacement parameters (Å^2^)

**Table:**

	*x*	*y*	*z*	*U*_iso_*/*U*_eq_	Occ. (<1)
Co1	0.62443 (4)	0.72407 (5)	0.45042 (3)	0.0370 (2)	
C1	0.6243 (3)	0.7598 (4)	0.2917 (2)	0.0483 (11)	
H1A	0.6235	0.8444	0.2989	0.058*	
C2	0.6240 (3)	0.7152 (4)	0.2223 (2)	0.0570 (12)	
H2A	0.6231	0.7697	0.1836	0.068*	
C3	0.6250 (3)	0.5917 (5)	0.2099 (2)	0.0564 (12)	
H3A	0.6252	0.5610	0.1631	0.068*	
C4	0.6257 (3)	0.5138 (4)	0.2681 (2)	0.0512 (11)	
H4A	0.6268	0.4292	0.2610	0.061*	
C5	0.6249 (3)	0.5610 (4)	0.3374 (2)	0.0418 (9)	
C6	0.6225 (3)	0.4894 (4)	0.4023 (2)	0.0415 (10)	
H6A	0.6220	0.4039	0.4027	0.050*	
C7	0.6191 (3)	0.6008 (4)	0.5726 (2)	0.0390 (9)	
C8	0.6173 (3)	0.5700 (4)	0.6503 (2)	0.0384 (9)	
C9	0.6228 (3)	0.4484 (4)	0.6718 (2)	0.0490 (11)	
H9A	0.6266	0.3870	0.6373	0.059*	
C10	0.6229 (3)	0.4173 (4)	0.7431 (2)	0.0526 (11)	
H10A	0.6271	0.3352	0.7570	0.063*	
C11	0.6168 (3)	0.5066 (4)	0.7938 (2)	0.0479 (11)	
C12	0.6086 (3)	0.6291 (4)	0.7744 (2)	0.0579 (12)	
H12A	0.6029	0.6895	0.8093	0.069*	
C13	0.6090 (3)	0.6601 (4)	0.7025 (2)	0.0530 (11)	
H13A	0.6036	0.7421	0.6887	0.064*	
C14	0.8270 (3)	0.6589 (4)	0.4748 (2)	0.0476 (10)	
H14A	0.8098	0.5766	0.4775	0.057*	
C15	0.9209 (3)	0.6903 (5)	0.4849 (2)	0.0561 (12)	
H15A	0.9659	0.6295	0.4952	0.067*	
C16	0.9482 (3)	0.8086 (5)	0.4799 (3)	0.0600 (12)	
H16A	1.0118	0.8296	0.4860	0.072*	
C17	0.8803 (3)	0.8982 (4)	0.4656 (2)	0.0565 (12)	
H17A	0.8977	0.9803	0.4621	0.068*	
C18	0.7860 (3)	0.8647 (4)	0.4565 (2)	0.0440 (10)	
C19	0.7070 (3)	0.9470 (4)	0.4422 (2)	0.0483 (10)	
H19A	0.7140	1.0311	0.4355	0.058*	
C20	0.4752 (3)	0.8648 (4)	0.4255 (2)	0.0424 (10)	
C21	0.3756 (3)	0.9004 (4)	0.4079 (2)	0.0441 (10)	
C22	0.3531 (4)	1.0132 (4)	0.3757 (3)	0.0670 (14)	
H22A	0.4010	1.0688	0.3677	0.080*	
C23	0.2620 (4)	1.0433 (5)	0.3557 (3)	0.0745 (16)	
H23A	0.2480	1.1185	0.3335	0.089*	
C24	0.1905 (3)	0.9620 (5)	0.3684 (3)	0.0631 (13)	
C25	0.2097 (3)	0.8526 (4)	0.4026 (3)	0.0611 (13)	
H25A	0.1608	0.7997	0.4126	0.073*	
C26	0.3019 (3)	0.8217 (4)	0.4219 (2)	0.0520 (11)	
H26A	0.3152	0.7469	0.4448	0.062*	
N2	0.6211 (2)	0.5548 (3)	0.45984 (16)	0.0360 (7)	
N3	0.6194 (2)	0.5074 (3)	0.52737 (16)	0.0398 (8)	
N4	0.7595 (2)	0.7441 (3)	0.46134 (16)	0.0396 (8)	
N5	0.6262 (2)	0.8936 (3)	0.43946 (16)	0.0397 (8)	
N6	0.5418 (2)	0.9507 (3)	0.42431 (19)	0.0464 (9)	
N1	0.6257 (2)	0.6854 (3)	0.34889 (17)	0.0398 (8)	
O2	0.4919 (2)	0.7504 (2)	0.43905 (15)	0.0430 (7)	
O1	0.6200 (2)	0.7154 (2)	0.55317 (14)	0.0421 (7)	
Cl2	0.07430 (10)	0.99916 (16)	0.34145 (10)	0.0988 (6)	
Cl1	0.61734 (11)	0.46595 (13)	0.88446 (6)	0.0751 (4)	
N7	0.6640 (3)	0.2922 (4)	0.0902 (3)	0.0703 (12)	
O3	0.6977 (6)	0.3291 (6)	0.1463 (4)	0.199 (3)	
O4	0.6469 (4)	0.3662 (5)	0.0448 (3)	0.134 (2)	
O5	0.6521 (4)	0.1837 (4)	0.0843 (3)	0.1204 (17)	
O6	0.6525 (4)	0.0520 (6)	0.2242 (4)	0.216 (4)	
H61	0.6556	0.0630	0.1791	0.259*	
H62	0.6562	0.1131	0.2529	0.259*	
O7	0.5975 (6)	0.1822 (9)	0.3446 (9)	0.193 (7)	0.50
H72	0.5956	0.1360	0.3814	0.231*	0.50
H71	0.5465	0.1745	0.3180	0.231*	0.50

### Atomic displacement parameters (Å^2^)

**Table:**

	*U*^11^	*U*^22^	*U*^33^	*U*^12^	*U*^13^	*U*^23^
Co1	0.0475 (4)	0.0276 (3)	0.0361 (3)	−0.0045 (2)	0.0047 (2)	0.0015 (2)
C1	0.056 (3)	0.046 (3)	0.043 (2)	0.000 (2)	0.005 (2)	0.010 (2)
C2	0.069 (3)	0.060 (3)	0.041 (3)	−0.002 (2)	0.000 (2)	0.012 (2)
C3	0.069 (3)	0.064 (3)	0.036 (2)	−0.001 (2)	0.002 (2)	−0.003 (2)
C4	0.061 (3)	0.051 (3)	0.041 (2)	−0.002 (2)	0.005 (2)	−0.008 (2)
C5	0.046 (2)	0.038 (2)	0.041 (2)	−0.0017 (18)	0.0039 (18)	0.0005 (18)
C6	0.050 (3)	0.028 (2)	0.046 (2)	−0.0014 (17)	0.0017 (19)	−0.0037 (18)
C7	0.037 (2)	0.041 (2)	0.040 (2)	−0.0072 (17)	0.0034 (17)	0.0026 (18)
C8	0.041 (2)	0.036 (2)	0.037 (2)	−0.0077 (17)	−0.0002 (17)	−0.0015 (17)
C9	0.072 (3)	0.038 (2)	0.037 (2)	0.003 (2)	0.005 (2)	−0.0026 (19)
C10	0.080 (3)	0.037 (2)	0.042 (2)	0.006 (2)	0.008 (2)	0.0050 (19)
C11	0.055 (3)	0.051 (3)	0.037 (2)	−0.003 (2)	−0.0004 (19)	0.004 (2)
C12	0.081 (3)	0.050 (3)	0.044 (3)	−0.001 (2)	0.010 (2)	−0.010 (2)
C13	0.074 (3)	0.042 (3)	0.043 (3)	−0.005 (2)	0.006 (2)	−0.001 (2)
C14	0.058 (3)	0.041 (3)	0.043 (2)	0.002 (2)	0.003 (2)	0.0096 (19)
C15	0.048 (3)	0.065 (3)	0.055 (3)	0.000 (2)	−0.001 (2)	0.008 (2)
C16	0.049 (3)	0.068 (3)	0.062 (3)	−0.009 (2)	−0.002 (2)	0.001 (3)
C17	0.060 (3)	0.051 (3)	0.059 (3)	−0.020 (2)	0.004 (2)	−0.005 (2)
C18	0.056 (3)	0.036 (2)	0.040 (2)	−0.009 (2)	0.0058 (19)	−0.0025 (18)
C19	0.063 (3)	0.034 (2)	0.049 (3)	−0.010 (2)	0.008 (2)	−0.0012 (19)
C20	0.055 (3)	0.031 (2)	0.042 (2)	0.0000 (19)	0.0076 (19)	0.0006 (18)
C21	0.051 (3)	0.031 (2)	0.051 (3)	−0.0004 (18)	0.0085 (19)	−0.0012 (19)
C22	0.056 (3)	0.048 (3)	0.097 (4)	0.002 (2)	0.001 (3)	0.017 (3)
C23	0.060 (3)	0.059 (3)	0.103 (4)	0.009 (3)	−0.001 (3)	0.027 (3)
C24	0.051 (3)	0.062 (3)	0.075 (3)	0.009 (2)	0.002 (2)	0.000 (3)
C25	0.053 (3)	0.050 (3)	0.082 (4)	−0.007 (2)	0.016 (2)	−0.005 (3)
C26	0.058 (3)	0.038 (2)	0.061 (3)	0.007 (2)	0.012 (2)	−0.001 (2)
N2	0.0427 (19)	0.0295 (17)	0.0358 (18)	−0.0042 (14)	0.0018 (14)	0.0020 (14)
N3	0.054 (2)	0.0321 (18)	0.0338 (18)	−0.0053 (15)	0.0054 (15)	0.0006 (14)
N4	0.052 (2)	0.0350 (19)	0.0312 (17)	−0.0071 (15)	−0.0004 (15)	0.0032 (14)
N5	0.047 (2)	0.0315 (18)	0.0403 (19)	−0.0031 (16)	0.0040 (15)	0.0002 (14)
N6	0.054 (2)	0.0316 (19)	0.054 (2)	−0.0037 (17)	0.0091 (17)	0.0000 (16)
N1	0.045 (2)	0.0368 (19)	0.0374 (18)	−0.0040 (15)	0.0019 (14)	0.0046 (15)
O2	0.0511 (17)	0.0291 (15)	0.0494 (17)	−0.0049 (12)	0.0083 (13)	0.0005 (12)
O1	0.0580 (18)	0.0281 (15)	0.0407 (15)	−0.0045 (12)	0.0066 (13)	−0.0002 (12)
Cl2	0.0549 (9)	0.1056 (13)	0.1344 (15)	0.0108 (8)	−0.0029 (9)	0.0189 (10)
Cl1	0.1181 (12)	0.0727 (9)	0.0344 (6)	0.0010 (8)	0.0045 (6)	0.0036 (6)
N7	0.097 (4)	0.053 (3)	0.058 (3)	−0.008 (2)	−0.013 (2)	−0.015 (2)
O3	0.321 (10)	0.144 (6)	0.126 (5)	−0.035 (6)	−0.016 (6)	−0.042 (4)
O4	0.151 (5)	0.097 (4)	0.147 (5)	−0.023 (3)	−0.030 (3)	0.062 (4)
O5	0.156 (5)	0.063 (3)	0.136 (4)	−0.002 (3)	−0.031 (3)	−0.022 (3)
O6	0.194 (6)	0.222 (8)	0.216 (7)	−0.131 (6)	−0.096 (5)	0.133 (6)
O7	0.077 (6)	0.087 (7)	0.41 (2)	−0.015 (5)	−0.043 (9)	0.138 (10)

### Geometric parameters (Å, °)

**Table:**

Co1—N2	1.850 (3)	C14—H14A	0.9300
Co1—N5	1.855 (3)	C15—C16	1.349 (7)
Co1—O2	1.899 (3)	C15—H15A	0.9300
Co1—O1	1.914 (3)	C16—C17	1.384 (7)
Co1—N4	1.926 (4)	C16—H16A	0.9300
Co1—N1	1.931 (3)	C17—C18	1.385 (6)
C1—N1	1.334 (5)	C17—H17A	0.9300
C1—C2	1.375 (6)	C18—N4	1.369 (5)
C1—H1A	0.9300	C18—C19	1.444 (6)
C2—C3	1.363 (6)	C19—N5	1.283 (5)
C2—H2A	0.9300	C19—H19A	0.9300
C3—C4	1.372 (6)	C20—O2	1.287 (4)
C3—H3A	0.9300	C20—N6	1.331 (5)
C4—C5	1.385 (6)	C20—C21	1.479 (6)
C4—H4A	0.9300	C21—C22	1.392 (6)
C5—N1	1.369 (5)	C21—C26	1.392 (6)
C5—C6	1.438 (6)	C22—C23	1.359 (7)
C6—N2	1.284 (5)	C22—H22A	0.9300
C6—H6A	0.9300	C23—C24	1.380 (7)
C7—O1	1.297 (5)	C23—H23A	0.9300
C7—N3	1.318 (5)	C24—C25	1.367 (7)
C7—C8	1.483 (5)	C24—Cl2	1.736 (5)
C8—C9	1.381 (6)	C25—C26	1.374 (6)
C8—C13	1.388 (6)	C25—H25A	0.9300
C9—C10	1.366 (6)	C26—H26A	0.9300
C9—H9A	0.9300	N2—N3	1.357 (4)
C10—C11	1.359 (6)	N5—N6	1.362 (5)
C10—H10A	0.9300	N7—O4	1.176 (6)
C11—C12	1.383 (6)	N7—O3	1.185 (7)
C11—Cl1	1.738 (4)	N7—O5	1.197 (6)
C12—C13	1.376 (6)	O6—H61	0.8498
C12—H12A	0.9300	O6—H62	0.8501
C13—H13A	0.9300	O7—H72	0.8501
C14—N4	1.344 (5)	O7—H71	0.8499
C14—C15	1.376 (6)		
			
N2—Co1—N5	178.95 (14)	C16—C15—C14	120.5 (5)
N2—Co1—O2	97.39 (12)	C16—C15—H15A	119.7
N5—Co1—O2	81.92 (13)	C14—C15—H15A	119.7
N2—Co1—O1	81.59 (12)	C15—C16—C17	119.1 (4)
N5—Co1—O1	99.20 (12)	C15—C16—H16A	120.5
O2—Co1—O1	90.78 (12)	C17—C16—H16A	120.5
N2—Co1—N4	97.76 (13)	C16—C17—C18	119.4 (4)
N5—Co1—N4	82.94 (14)	C16—C17—H17A	120.3
O2—Co1—N4	164.81 (13)	C18—C17—H17A	120.3
O1—Co1—N4	90.33 (13)	N4—C18—C17	120.8 (4)
N2—Co1—N1	82.97 (13)	N4—C18—C19	113.1 (4)
N5—Co1—N1	96.22 (13)	C17—C18—C19	126.1 (4)
O2—Co1—N1	90.24 (13)	N5—C19—C18	114.0 (4)
O1—Co1—N1	164.53 (13)	N5—C19—H19A	123.0
N4—Co1—N1	92.71 (13)	C18—C19—H19A	123.0
N1—C1—C2	122.0 (4)	O2—C20—N6	124.1 (4)
N1—C1—H1A	119.0	O2—C20—C21	117.1 (3)
C2—C1—H1A	119.0	N6—C20—C21	118.7 (4)
C3—C2—C1	120.4 (4)	C22—C21—C26	118.1 (4)
C3—C2—H2A	119.8	C22—C21—C20	120.8 (4)
C1—C2—H2A	119.8	C26—C21—C20	121.1 (4)
C2—C3—C4	118.4 (4)	C23—C22—C21	120.9 (5)
C2—C3—H3A	120.8	C23—C22—H22A	119.5
C4—C3—H3A	120.8	C21—C22—H22A	119.5
C3—C4—C5	120.1 (4)	C22—C23—C24	119.8 (5)
C3—C4—H4A	119.9	C22—C23—H23A	120.1
C5—C4—H4A	119.9	C24—C23—H23A	120.1
N1—C5—C4	120.7 (4)	C25—C24—C23	120.9 (5)
N1—C5—C6	113.9 (3)	C25—C24—Cl2	119.3 (4)
C4—C5—C6	125.4 (4)	C23—C24—Cl2	119.8 (4)
N2—C6—C5	113.5 (4)	C24—C25—C26	119.2 (4)
N2—C6—H6A	123.2	C24—C25—H25A	120.4
C5—C6—H6A	123.2	C26—C25—H25A	120.4
O1—C7—N3	124.3 (3)	C25—C26—C21	121.1 (4)
O1—C7—C8	119.3 (3)	C25—C26—H26A	119.5
N3—C7—C8	116.5 (3)	C21—C26—H26A	119.5
C9—C8—C13	118.7 (4)	C6—N2—N3	124.0 (3)
C9—C8—C7	119.5 (4)	C6—N2—Co1	118.1 (3)
C13—C8—C7	121.8 (4)	N3—N2—Co1	117.9 (2)
C10—C9—C8	120.8 (4)	C7—N3—N2	107.2 (3)
C10—C9—H9A	119.6	C14—N4—C18	118.5 (4)
C8—C9—H9A	119.6	C14—N4—Co1	129.2 (3)
C11—C10—C9	119.9 (4)	C18—N4—Co1	112.2 (3)
C11—C10—H10A	120.1	C19—N5—N6	124.9 (4)
C9—C10—H10A	120.1	C19—N5—Co1	117.7 (3)
C10—C11—C12	121.1 (4)	N6—N5—Co1	117.2 (2)
C10—C11—Cl1	119.5 (3)	C20—N6—N5	107.0 (3)
C12—C11—Cl1	119.4 (3)	C1—N1—C5	118.4 (4)
C13—C12—C11	118.8 (4)	C1—N1—Co1	130.1 (3)
C13—C12—H12A	120.6	C5—N1—Co1	111.5 (2)
C11—C12—H12A	120.6	C20—O2—Co1	109.6 (2)
C12—C13—C8	120.6 (4)	C7—O1—Co1	109.0 (2)
C12—C13—H13A	119.7	O4—N7—O3	116.5 (6)
C8—C13—H13A	119.7	O4—N7—O5	126.2 (5)
N4—C14—C15	121.7 (4)	O3—N7—O5	117.3 (6)
N4—C14—H14A	119.2	H61—O6—H62	120.0
C15—C14—H14A	119.2	H72—O7—H71	109.5

### Hydrogen-bond geometry (Å, °)

**Table:**

*D*—H···*A*	*D*—H	H···*A*	*D*···*A*	*D*—H···*A*
O6—H61···O5	0.85	2.19	2.964 (8)	151
O6—H62···O7	0.85	2.09	2.804 (16)	141
O7—H72···N6^i^	0.85	2.32	3.053 (12)	145
C14—H14A···O4^ii^	0.93	2.41	3.229 (7)	146
C17—H17A···O5^iii^	0.93	2.46	3.263 (7)	145

Symmetry codes: (i) *x*, *y*−1, *z*; (ii) −*x*+3/2, *y*, −*z*+1/2; (iii) −*x*+3/2, *y*+1, −*z*+1/2.
